# Wheat and barley can increase grain yield in shade through acclimation of physiological and morphological traits in Mediterranean conditions

**DOI:** 10.1038/s41598-019-46027-9

**Published:** 2019-07-02

**Authors:** M. G. Arenas-Corraliza, V. Rolo, M. L. López-Díaz, G. Moreno

**Affiliations:** INDEHESA Research Institute, University of Extremadura, Avenida Virgen del Puerto, 2, 10600 Plasencia (Cáceres), Spain

**Keywords:** Light responses, Photosynthesis, Heat, Light stress

## Abstract

Major cereal yields are expected to decline significantly in coming years due to the effects of climate change temperature rise. Agroforestry systems have been recognized as a useful land management strategy that could mitigate these effects through the shelter provided by trees, but it is unclear how shade affects cereal production. Most cereal species and cultivars have been selected for full light conditions, making it necessary to determine those able to acclimate to low irradiance environments and the traits that drive this acclimation. A greenhouse experiment was conducted in central Spain to assess the photosynthetic response, leaf morphology and grain yield of nine cultivars of winter wheat (*Triticum aestivum* L.) and barley (*Hordeum vulgare* L.) at three levels of photosynthetic active radiation (100%, 90% and 50%). Cultivars were selected according to three different precocity categories and were widely used in the studied area. The main objective was to assess whether the species and cultivars could acclimate to partial shade through physiological and morphological acclimations and thus increase their grain yield for cultivation in agroforestry systems. Both species increased grain yield by 19% in shade conditions. However, they used different acclimation strategies. Barley mostly performed a physiological acclimation, while wheat had a major morphological adjustment under shaded environment. Barley had lower dark respiration (42%), lower light compensation point (73%) and higher maximum quantum yield (48%) than wheat in full light conditions, revealing that it was a more shade-tolerant species than wheat. In addition, to acclimate to low irradiance conditions, barley showed a 21% reduction of the carotenoids/chlorophyll ratio in the lowest irradiance level compared to 100% light availability and adjusted the chlorophyll a/b ratio, photosystem II quantum efficiency, electron transport rate and non-photochemical quenching to shade conditions. On the other hand, wheat showed a 48% increase in single leaf area in the 50% irradiance level than in full light to maximize light capture. Our results showed that current commercialized wheat and barley cultivars had sufficient plasticity for adaptation to shade, supporting tree presence as a tool to reduce the negative effects of climate change.

## Introduction

Global food production has risen as never before in the last 60 years due to agricultural expansion and intensification. However, it is increasingly questioned if global food production will meet future demands. Crop yields have stagnated in recent years, while human population continues to increase at the same rate. Climate change and scarcity of new arable land have been indicated as the main drivers of the widening gap between food production and demand^1^. For major crops like barley and wheat, two of the world’s four most important cereal crops, grain yield is expected to decrease 15–30% as a result of 2 °C warming forecast for 2040^2,3^. This makes it necessary to design more climate-resilient and sustainable systems.

Ecological intensification, where crop yield can increase through better use of resources, has been postulated as a possible solution^4,5^. Agroforestry systems are an example of this concept^6^. Agroforestry has been recognized as a climate change adaptation tool, because trees help to regulate the climate beneath them by reducing extreme temperatures, providing wind shelter and reducing evaporation from the soil surface^7,8^. Integrating trees with crops (i.e., silvoarable agroforestry) has been shown to have a number of environmental benefits over conventional agriculture^9,10^, including a buffering effect on extreme temperatures^11^. However, tree presence can affect negatively crop yields^12,13^ because of shading or competition for soil resources. Greater understanding is required of how low irradiation due to tree presence affects the functioning of major crops and, therefore, their yield, before silvoarable systems can be implemented as an ecological intensification strategy against foreseen climate change. It is uncertain how cereal cultivars will cope with tree shade in silvoarable systems, because most cultivars have traditionally been selected for full light conditions.

To overcome the lack of knowledge about how low irradiance conditions will affect current cultivars, selection programs are needed to identify shade-adapted cereal species and cultivars for cultivation in agroforestry systems, based on analysis of functional plant traits that determine crop yield. To our knowledge no research has been conducted on photosynthetic low irradiance acclimation of wheat and barley in Mediterranean conditions, where average annual irradiance is higher than in the rest of Europe (https://solargis.info/). In the Mediterranean area, irradiance levels are usually in excess of plant requirements, and the main photosynthetic constraints are temperature and drought^14^. Recent studies in the Mediterranean area, where climate change consequences are expected to be more pronounced^15^, have shown a negative effect of tree presence on winter wheat grain yield^16,17^. However, there are mixed results when considering other areas with temperate^11^ and Mediterranean climate^18^, so the effect of shade on wheat grain yield remains unclear. Less is known about how shade can affect barley in the Mediterranean area. Barley has been cultivated in a wider range than most other crop species^19^ because it is a stress-tolerant crop. Furthermore, barley under low irradiance is able to change its photosynthetic performance in an acclimation process^20^, making it a potential target for studies on agroforestry shaded environments in a global climate change context.

In addition to reducing available light, trees can negatively affect crops because of competition for soil resources (water and nutrients). Water and nutrient competition can be remedied by adoption of appropriate agronomic practices, but low irradiance cannot be modified and must be managed through selection programs for acclimated cultivars. In breeding programs for complex agrosystems (i.e., agroforestry systems), selection based on functional trait screening (e.g., physiology and morphology) could be more relevant than selection based on genotype screening, as functional plant traits are key factors in the understanding of ecosystem-vegetation functioning^21^. In the case of low irradiance acclimation, it is important to identify whether there is sufficient variability among crop species and within their cultivars that could help to identify shade-acclimated genotypes.

The aim of this research was to assess the extent of shade acclimations of winter wheat and barley and their cultivars. We determined if photosynthetic and morphological traits are involved in shade acclimation and influence grain yield. Our hypotheses were:

1. *Shade reduces the grain yield of wheat but not of barley*, *which is able to withstand low irradiance conditions*.
2. *Barley is able to photosynthetically and morphologically adapt to shaded environments*, *since it is known to be a more stress-tolerant crop than wheat*.
3. *Within the cultivars of each species*, *there is no enough variability for selection programs in physiological and morphological traits that could drive to an increase of grain yield in shade conditions*.

## Material and Methods

### Plant material and experimental design

An open air greenhouse experiment was conducted at the Organic and Mountain Agriculture Research Center, Plasencia (40° 1′N, 6° 6′W), Spain, in the 2016–2017 growing season (November-June). The study comprised nine different cultivars of winter barley (*Hordeum vulgare* L.) and nine of winter wheat (*Triticum aestivum* L.). Cultivars were selected to span three categories of precocity (flowering dates): very early, early and medium. Each category included three cultivars of each species. Barley cultivars were named B1 to B9, representing the cultivars Hispanic, Lavanda and Luzia (very early), Kalea, Lagalia and Carolina (early) and Meseta, Ibaiona and Crescendo (medium). Wheat cultivars were named W1 to W9, representing the cultivars Nogal, Nudel and Tocayo (very early), Algoritmo, Paledor and Solehio (early) and Toskani, Somontano and Nemo (medium). Cultivar seeds were provided by CICYTEX (http://cicytex.juntaex.es/en/), which collaborates with the Group for the Evaluation of New Varieties for Extensive Crops in Spain (GENVCE: http://www.genvce.org/).

The experiment was a split design with irradiance as main block and cultivars as sub-block, with six replicate pots per cultivar of each species in each irradiance treatment (n = 6 for cultivars and n = 54 for species). Three irradiance levels were set for Photosynthetically Active Radiation (PAR): 100%, 90% and 50% by nets in different open greenhouse structures. The environmental conditions in each irradiance structure were recorded at the cereal canopy level (Table 1) by 2 dataloggers (PCE-H71N Data logger, PCE Instruments, Holding GmbH Inc., Hamburg, Germany). Nets were placed 1.5 m above plants to ensure sufficient ventilation. The material of the nets was green polyethylene. Mesh sizes were 2.25 cm^2^, 0.0075 cm^2^ and 0.0026 cm^2^ for 100%, 90% and 50% nets respectively. The 100% irradiance coverage was set up to avoid bird damage and did not reduced PAR compared to full light conditions. The mean maximum PAR values achieved under the nets 100%, 90% and 50% were 1680, 1390 and 965 µmol m^−2^ s^−1^ respectively in spring and mimic shading levels potentially found in different stages in silvoarable systems with deciduous trees as walnut and poplar^16^. Each block was completely covered with nets from the start of booting (7 April 2017), coinciding with the leaf sprout of deciduous trees (common trees in silvoarable systems in the Mediterranean area), to maturity (15 June 2017). In each pot (13 × 13 × 17 cm), four seeds were sown in November 18^th^ 2016. To avoid stem breakages, training rods and strings were installed around the plants before booting. The soil mixture comprised three parts black peat, one-part sand and one-part perlite, with soil water capacity 119% and pH 5.8. All pots were fertilized in November 2016 with 58 kg N ha^−1^, 100 kg P_2_O_5_ ha^−1^ and 58 kg K_2_O ha^−1^. In February 2017, 200 kg N ha^−1^ was added to each pot with 46% urea. All pots were regularly irrigated following the indications of a humidity probe to maintain soil water capacity above 50% and avoid soil water stress.

**Table 1:** Mean values of temperature, relative humidity and PAR in the different irradiance levels in the anthesis period (2017/04/23).

Irradiance (%)	Temperature (°C)	Relative humidity (%)	PAR (µmol (photon) m^−2^ s^−1^)
100	22.40 ± 0.49	40.63 ± 0.25	1182.09 ± 48.39
90	23.67 ± 0.23	40.32 ± 0.025	897.95 ± 46.75
50	21.98 ± 0.41	41.13 ± 1.35	630.60 ± 35.73

Data represents means ± S.E. Different letters indicate significant differences between irradiance levels (*P* < *0*.*05*).

### Grain yield

When cereal plants reached ripening, all pots were harvested (June 14^th^ 2017) using hand clippers. Plants were dried at 60 °C to constant weight. All grains of all spikes per pot were counted and weighed and grain yield was reported in g m^−2^.

### Photosynthesis parameters

Photosynthetic light response curves were measured during the flowering period in the middle part of the first leaf in wheat (known as the flag leaf, because it is the most important leaf for photosynthesis) and the second leaf in barley (the first leaf of this species is too small to measure). Measurements were made in one plant per pot of each species in the 100% irradiance treatment, when flowering was 50% complete in each cultivar. Curves were obtained using a portable infra-red gas analyzer (IRGA) (LCpro + , ADC Bioscientific Ltd., Hoddesdon, UK) set at 25 °C, with a CO_2_ concentration of 400 µl L^−1^ and a relative humidity of 65%. Leaves were first adapted to dark for 30 minutes, then exposed to increasing PAR intensities: 0, 176, 352, 616, 880, 1320 and 1584 µmol photons m^−2^ s^−1^ for 5 minutes, respectively. Net photosynthesis light-response curves were fitted according to the hyperbolic rectangular model, using the Solver function of Microsoft Excel 2013^22^. Five parameters of the hyperbolic rectangular model were used to compare photosynthetic behavior between species: dark respiration (R_D_); light compensation point (I_comp_), defined at the PAR level where net photosynthesis is null; maximum net photosynthesis (PN_max_); light saturation point beyond which there is no significant change in net photosynthesis (I_max_); and maximum quantum yield (Φ), as the derivate of the curve in the range between I_comp_ and I = 200 μmol photons m^−2^ s^−1^.

### Chlorophyll fluorescence

Rapid-light response curves of variable chlorophyll fluorescence were measured after including the three irradiance treatments (100%, 90% and 50%), using a modulated chlorophyll fluorometer (OS5p + , Opti-Sciences, Inc., Hudson, USA). Leaves were first dark-adapted for 30 minutes and then exposed to increasing actinic light intensities: 120, 494, 1639, 2192 and 2557 µmol photons m^−2^ s^−1^ in 15 second intervals. Steady state fluorescence ($$Fs)$$, maximum light-adapted fluorescence $$({F}^{\text{'}}m)\,\,$$and minimal fluorescence of the light-adapted state $$(Fo\text{'})$$ were determined to calculate photosystem II (PSII) quantum efficiency (Φ_PSII_) as:

$$((F\text{'}\,m-Fs))/(F\text{'}\,m)$$

Non-photochemical quenching (NPQ) was calculated by the expression:

$$((Fm-F\text{'}\,m))/F\text{'}m$$

Electron Transport Rate (ETR) was determined by:

$${{\rm{\Phi }}}_{PSII}\cdot PAR\cdot 0.84\cdot 0.5$$

where PAR refers to photosynthetically active radiation, 0.84 to leaf absorption coefficient^23^ and 0.5 to the equal distribution of absorbed light by PSI and PSII.

### Photosynthetic pigments

For rapid assessment of photosynthetic pigment content with minimum damage to leaves, SPAD units were taken in three parts of one leaf per pot in each irradiance treatment using a SPAD meter (SPAD-502 Plus, Konica Minolta Holdings, Inc.). To calibrate SPAD readings, nine fresh leaves of each species (three per light treatment) were used to determine the photosynthetic pigments. Extraction was performed by grinding 100 mg fresh matter in 10 ml acetone 80% (v/v). the resulting mixture was stored in photoprotective tubes at 4 °C and tubes were later centrifuged 15 minutes at 2500 rpm. The supernatant was stored in photoprotective tubes and analyzed later. The Chl a, Chl b, total Chl (a + b) and Carot (x + c) content were determined according to Lichtenthaler^24^ and correlated with SPAD units^25^. We fitted linear regressions separately for each species to relate SPAD units and photosynthetic pigments. Linear regressions fitted the data well both for barley (R^2^ = 0.893, 0.781, 0.881, 0.899 for each pigment respectively; p < 0.001) and wheat (R^2^ = 0.965, 0.918, 0.980, 0.745 for each pigment respectively; p < 0.001).

### Leaf mass area and single leaf area

Leaf mass area index was calculated as the ratio of leaf dry mass to leaf area, sampling one fresh leaf in each pot per cultivar. Fresh leaves were scanned and their area was calculated using *ImageJ* software (National Institute of Health, USA). These leaves were then dried at 60 °C to constant weight to obtain leaf dry mass.

### Statistical analysis

Because net photosynthetic curves are nonlinear, we fitted a GAM model to assess the overall differences in net photosynthesis between species. To test whether the parameters of the hyperbolic rectangular model differed between species, we fitted linear mixed models, including species as a fixed effect and cultivar as a random effect. To fit rapid-light response curves of fluorescence parameters, we also used GAMs. Fluorescence parameter models were fitted separately for each species, including irradiance as a predictor. We fitted linear mixed models, including species and irradiance and their interaction as fixed effects and cultivar as a random effect to analyze the response of photosynthetic pigments, leaf mass area, leaf area and grain yield at varying light levels for each species. Self-defined contrast was used to assess pairwise comparison between irradiance levels within species. For all linear mixed models, we computed the marginal (${R}_{m}^{2}$) and conditional (${R}_{c}^{2}$) coefficient of determination to assess the amount of variance explained by the fixed effects and the entire model including random effects, respectively^26^. To assess variability in cultivar response to light, we fitted linear regressions with grain yield, leaf mass area, total chlorophyll and non-photochemical quenching as response variables and light as a predictor for each cultivar separately. All analyses were performed using R version 3.5.3^27^.GAMs models were fit with “mgvc”^28^, linear mixed models with “lme4”^29^ pairwise comparisons were assessed with “multcomp”^30^ and coefficients of determination were computed with “MuMIn”^31^ R packages.

## Results

### Grain yield

Grain yield increased significantly in reduced irradiance treatments in both barley and wheat (P < 0.001, ${R}_{m}^{2}$ = 0.07, ${R}_{c}^{2}$ = 0.24) (Table 2). Barley showed a 19% increase in grain yield in the 50% light availability level compared to 90% and 100% irradiance and wheat had a 19% higher grain yield at both 90% and 50% irradiance levels compared to full light.

**Table 2:** Grain yield, photosynthetic pigments and morphological parameters of barley and wheat for the irradiance levels studied.

Species	Irradiance (%)	Grain yield (g m^−2^)	Chl a (mg g^−1^)	Chl b (mg g^−1^)	Carot (mg g^−1^)	Chl a/b	Carot/ Chl	LMA (mg cm^−2^)	LA (cm^−2^)
Barley	100	299.41 ± 10.22 **b**	1.13 ± 0.05 **b**	0.34 ± 0.01 **b**	0.37 ± 0.01 **b**	3.38 ± 0.01 **a**	0.28 ± 0.02 **a**	9.23 ± 0.22 **a**	2.30 ± 0.10
	90	304.65 ± 12.34 **b**	1.14 ± 0.05 **b**	0.34 ± 0.02 **b**	0.38 ± 0.01 **b**	3.38 ± 0.01 **a**	0.29 ± 0.02 **a**	8.13 ± 0.16 **ab**	2.30 ± 0.11
	50	355.84 ± 12.30 **a**	1.74 ± 0.05 **a**	0.52 ± 0.01 **a**	0.49 ± 0.01 **a**	3.34 ± 0.00 **b**	0.22 ± 0.00 **b**	7.87 ± 0.19 **b**	2.64 ± 0.12
Wheat	100	277.18 ± 11.27 **b**	1.47 ± 0.05 **b**	0.44 ± 0.01 **b**	0.44 ± 0.01 **b**	3.35 ± 0.00	0.24 ± 0.00	13.25 ± 0.48 **a**	3.81 ± 0.19 **c**
	90	322.54 ± 13.39 **a**	1.60 ± 0.04 **b**	0.48 ± 0.01 **b**	0.46 ± 0.01 **b**	3.35 ± 0.00	0.23 ± 0.00	10.01 ± 0.29 **b**	4.51 ± 0.18 **b**
	50	329.44 ± 11.50 **a**	2.01 ± 0.04 **a**	0.60 ± 0.01 **a**	0.54 ± 0.01 **a**	3.34 ± 0.00	0.21 ± 0.00	10.31 ± 0.26 **b**	5.65 ± 0.24 **a**

All chlorophyll samples were calculated from fresh material. Data represents means ± S.E. (n = 54). Different letters indicate significant differences between irradiance levels (*P* < *0*.*05*). Chl a: chlorophyll a; Chl b: chlorophyll b; Carot: carotenoids; Chl a/b: ratio of chlorophyll a to chlorophyll b; Carot/Chl: ratio of carotenoids to total chlorophyll (a + b); LMA: leaf mass area; LA: single leaf area.

### Physiological performance

Chlorophyll a and b increased at 50% irradiance compared to full light conditions in barley (54% and 53% respectively) and wheat (53% and 36% respectively) (Table 2), allowing plants to use the available light more efficiently. The Chl a/b ratio decreased a significant 1% at 50% irradiance in barley compared to full light conditions, while no acclimation was seen in wheat in this parameter. In addition, the Carot/Chl ratio decreased significantly in the 50% irradiance treatment in barley (21% less than in full light), and no shade effect was observed for wheat (Table 2).

**Figure 1:**
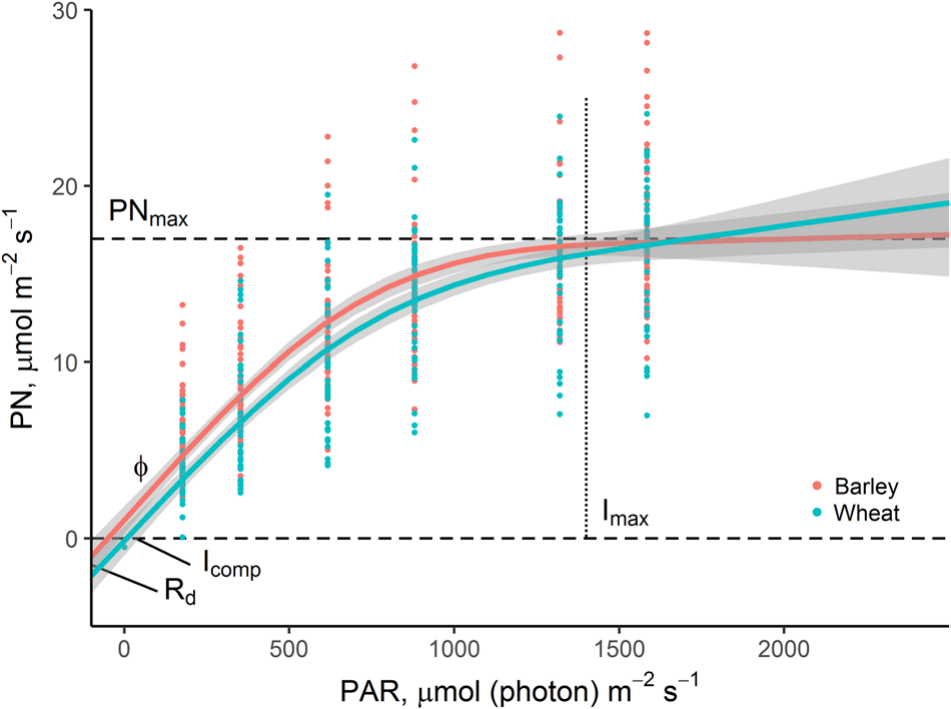
Net photosynthesis (PN) light-response curve (±95% CI) and associated parameters of barley and wheat plants grown at 100% irradiance level (n = 54). When CI are overlapped, there are no significant differences. R_D_: dark respiration [µmol (CO_2_) m^−2^ s^−1^]; I_comp_: light compensation point [µmol (photon) m^−2^ s^−1^]; PN_max_: maximum net photosynthesis [µmol m^−2^ s^−1^]; I_max_: light saturation point beyond which there is no significant increase in net photosynthesis [µmol (photon) m^−2^ s^−1^]; Φ_(*I*comp-*I*200)_: maximum quantum yield in the range between I_comp_ and I = 200 µmol (photon) m^−2^ s^−1^ [µmol (CO_2_) µmol (photon)^−1^]. Note that parameters values were calculated according to Lobo *et al*.^22^ and GAMs to assess the overall difference between species on the light-response curve and for visualization purposes.

Overall, wheat had lower net photosynthetic values than barley when considering the whole PAR gradient of the light response curve (GAM model, R^2^ = 0.75, *P* < *0*.*001*). However, these differences were mainly observed at low PAR values (Fig. 1). This agrees with the results of the hyperbolic rectangular mode fits, where there were significantly lower values of R_D_, I_comp_ and I_max_ in barley than in wheat. The signifcantly lower I_comp_ in barley (9.6 ± 1.5 [µmol (photon) m^−2^ s^−1^]) than in wheat (35.1 ± 6.6 [µmol (photon) m^−2^ s^−1^]) (P < 0.022, ${R}_{m}^{2}$ = 0.21, ${R}_{c}^{2}$ = 0.88) was a result of the also significantly lower R_D_ in barley (0.46 ± 0.07 [µmol (CO_2_) m^−2^ s^−1^]) compared with wheat (0.79 ± 0.08 [µmol (CO_2_) m^−2^ s^−1^]) (P = 0.022, ${R}_{m}^{2}$ = 0.17, ${R}_{c}^{2}$ = 0.57), which was quickly balanced by lower rates of photosynthesis. In addition, the lower I_max_ in barley (1807.7 ± 41.2 µmol (photon) m^−2^ s^−1^) allowed this species to maximize its net photosynthesis rate in relatively low light intensisties, while wheat continued to slightly raise its carbon assimilation to 1848.9 ± 60.8 µmol (photon) m^−2^ s^−1^, showing a greater need for light to reach its highest photosynthetic activity (i.e. I_max_). There was no difference in the maximum net photosynthesis between barley (18.3 ± 0.9) and wheat (17 ± 0.9). However, a strong difference was found in Φ_(*I*comp-*I*200)_, which was 48% higher in barley (Fig. 1) than in wheat (0.034 ± 0.002 and 0.023 ± 0.002) (P = 0.001, ${R}_{m}^{2}$ = 0.28, ${R}_{c}^{2}$ = 0.52), representing the initial slope of the light-response curve of net photosynthetic rate (i.e., the region limited by photochemical rather than biochemical reactions). Photosynthetic parameters showed a high variability among cultivars as indicated by the large differences between marginal (${R}_{m}^{2}$) and conditional (${R}_{c}^{2}$) coefficients of determination in all models.

**Figure 2:**
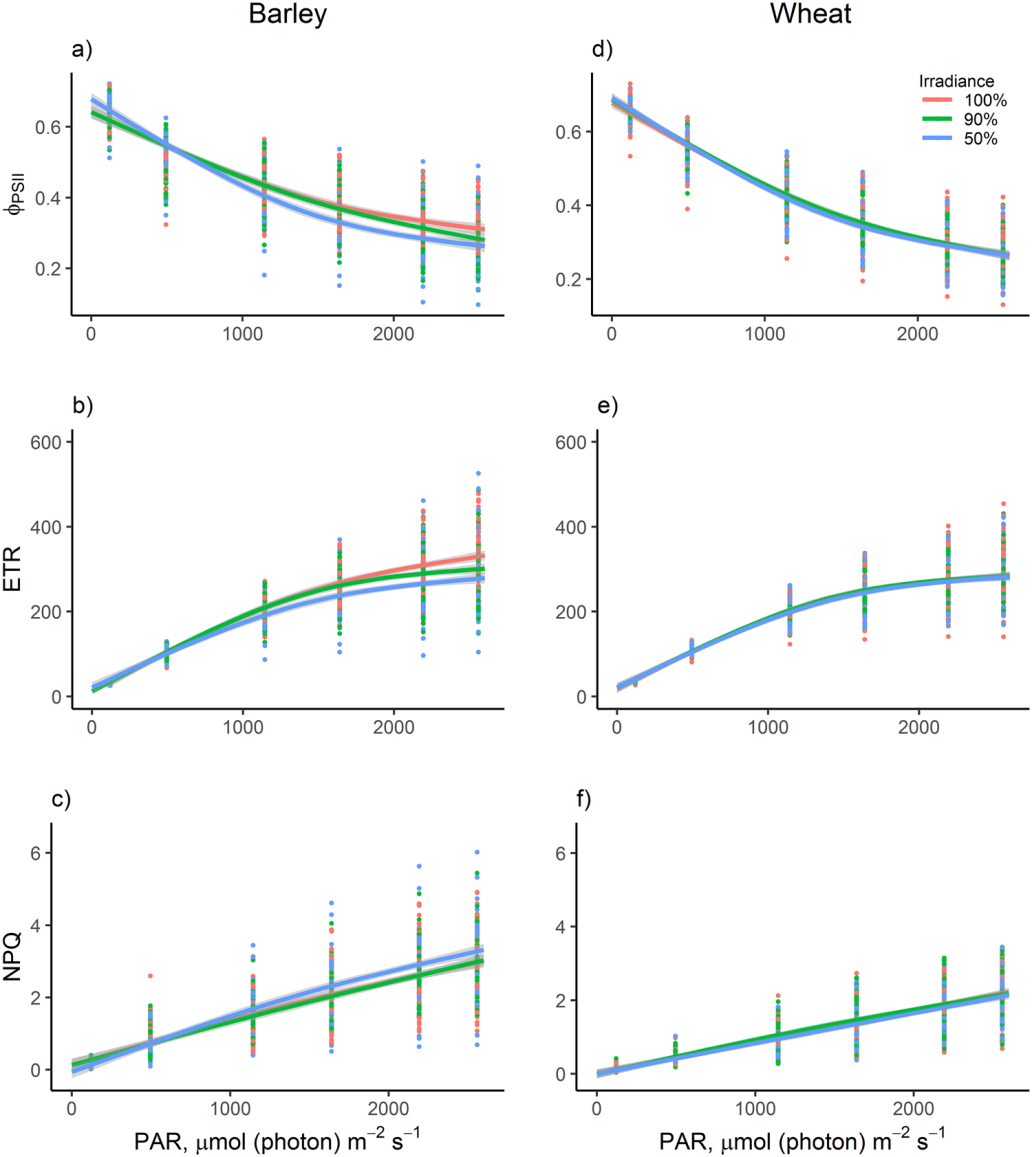
Rapid-light response curves (±95% CI) of photosystem II quantum efficiency (Φ_PSII_), electron transport rate (ETR) and non-photochemical quenching (NPQ) of barley (**a–c**) and wheat (**d–f**) grown in the irradiance conditions studied (100%, 90% and 50%) at different PAR intensities (n = 54).

Fluorescence parameters of the PSII (i.e., Φ_PSII_, ETR and NPQ) responded differently to changing irradiances for wheat and barley (Fig. 2). While no significant differences were detected for wheat, barley plants grown at 50% irradiance typically showed significant differences in Φ_PSII_, ETR and NPQ than plants grown at 90% (P = 0.011, P < 0.001 and P = 0.007, respectively) and 100% irradiance (P < 0.001, P < 0.001 and P = 0.019, respectively). Barley plants grown at 50% irradiance had higher Φ_PSII_ in low PAR values than those of plants grown at 90% and 100% irradiance. However, when exposed to high PAR intensities, barley grown at 50% irradiance showed lower Φ_PSII,_ (Fig. 2a) and ETR (Fig. 2b) and higher NPQ (Fig. 2c). Barley plants grown at 90% irradiance only showed significantly lower ETR values than plants grown at full light (P = 0.023).

### Morphological acclimation

A decrease in LMA in reduced irradiance treatments for both species barley and wheat compared to full light conditions was detected (maximum reductions of 15% in barley and 22% in wheat, LMA x species x irradiance, P < 0.001, ${R}_{m}^{2}$ = 0.41, ${R}_{c}^{2}$ = 0.41) (Table 2). In terms of single leaf surface (LA x species x irradiance, P < 0.001, ${R}_{m}^{2}$ = 0.51, ${R}_{c}^{2}$ = 0.65), for wheat, it was higher (48% and 18%) in the reduced irradiance levels of 90% and 50% respectively when compared to full light conditions (Table 2), in contrast with barley, which did not show this acclimation to shade. Marginal (${R}_{m}^{2}$) and conditional (${R}_{c}^{2}$) coefficients of determination showed similar values in both models, suggesting low variability of morphological parameters among cultivars.

### Cultivars plasticity

**Figure 3:**
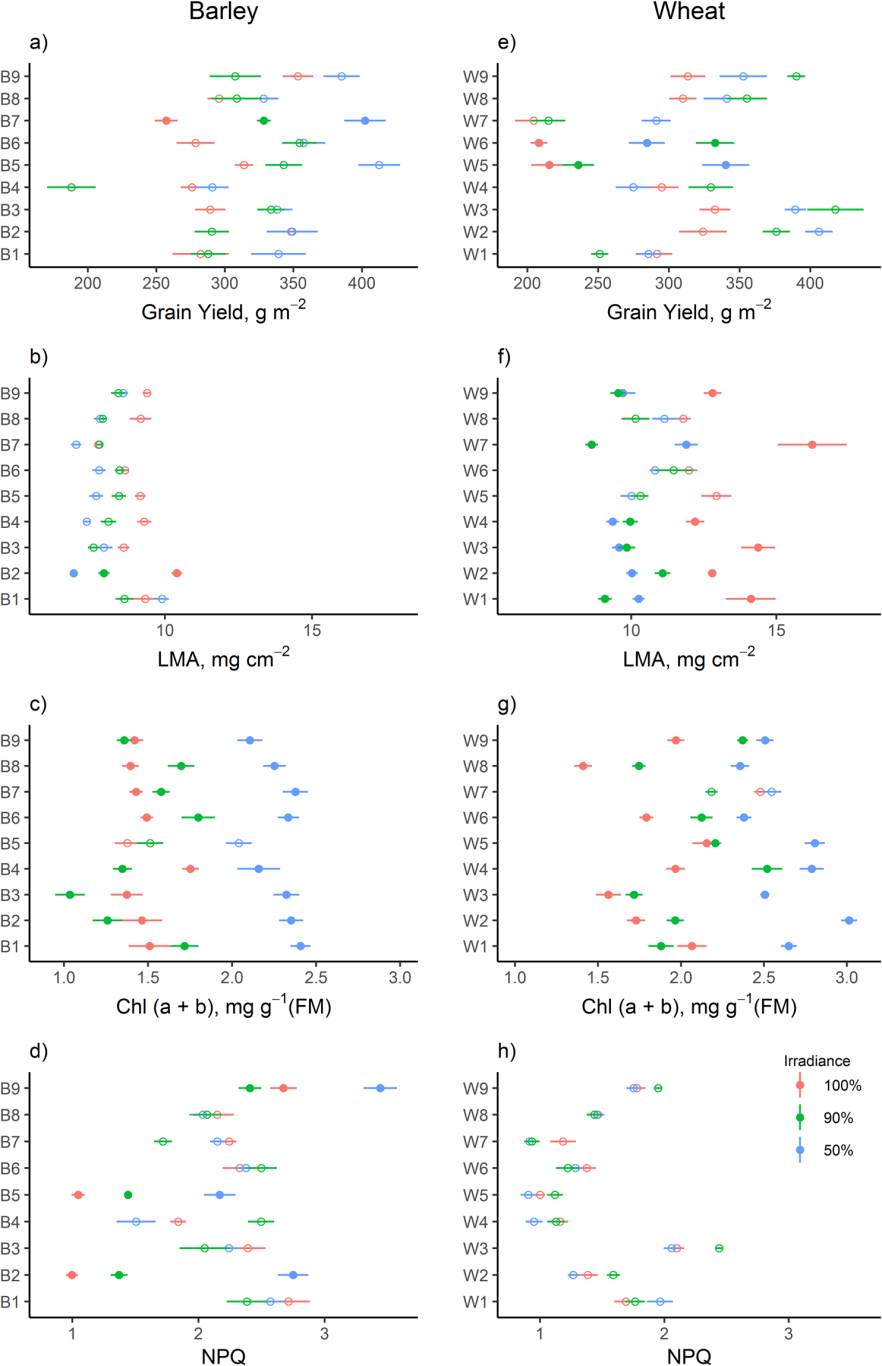
Grain yield and morphophysiological variables (±S.E.), including non-photochemical quenching (NPQ) at 1639 µmol photons m^−2^ s^−1^, of the cultivars studied for each species at different levels of irradiance (100%, 90% and 50%) (n = 6). Closed circles represent a significant effect of irradiance at *P* < *0*.*05*.

Depending on the parameter consider, cultivar performances ranged from no acclimation to considerable changes in shade in both species. The tendency of increasing grain yield under low irradiance observed at species level (Table 2) was also confirmed in cultivars, because most barley grain increases were at 50% irradiance and those in wheat were at 90%, although grain yield increases depended on the cultivars and were significant only for cultivars B7 (barley) and W5 and W6 (wheat) (Fig. 3). LMA showed higher variability in wheat than in barley (e.g 8.9% and 2.5% LMA coefficient of variation of wheat and barley, respectively), revealing that most wheat cultivars modify their LMA to adapt to different light environments (Fig. 3). This was also observed at species level (Table 2). However, some wheat cultivars revealed no acclimation to shade (W5, W6 and W8) in this parameter, indicating that some cultivars were more able than others to modify this morphological trait. For Chl (a + b), even though almost all cultivars of both barley and wheat increased their content at low irradiance levels, agreeing with the results at species level (Table 2), wheat did not show a pattern as clear as barley. Barley cultivars had almost the same Chl (a + b) content in 100% and in 50% irradiance levels, while wheat cultivars showed more dispersion in Chl (a + b) values in each irradiance conditions. Furthermore, as observed in both barley and wheat species (Fig. 2), barley cultivars revealed greater low-irradiance acclimation in NPQ than wheat, which showed no variation. Nevertheless, this adjustment was not as consistent in cultivars as at species level and was significant only in B2, B5 and B9 cultivars, while the others showed no acclimation. Wheat cultivars that had lower grain yield also had lower NPQ values, suggesting that non-photochemical quenching could be an interesting trait for cultivar selection. The precocity of the cultivars was not significant in any of the parameters studied.

## Discussion

### Both wheat and barley increased grain yield in shade

In contrast to our expectation, both species increased grain yield in shade as compared to full light conditions. Although wheat increased grain yield with shade compared to full light, it reached the same grain yield at 90% irradiance treatment as in the 50% irradiance level. In fact, Xu *et al*.^32^ also reported that grain yield did not increase significantly below 90% light availability. Contrary to our results, decreases in grain yield under 70–80% irradiance treatments were found in 35% lower solar radiation conditions^33^ than those in the Mediterranean (https://solargis.info/). However, barley increased grain yield significantly in the lowest irradiance level (i.e. 50% irradiance), according to the results reported under tree shade in a silvoarable system in the same Mediterranean conditions as our study^13^. All these results indicate that cereal species like barley and wheat grown in high irradiance conditions, such as those in the Mediterranean, can benefit from partial shade. Decreases in cereal production may be explained by lower solar radiation and/or competition for soil resources in agroforestry systems. Because this competition can be amended (with irrigation or fertilization) or avoided (decidious trees that sprout when cereals have completed most of their nutrient uptake), agroforestry could be a sustainable land-use system to help meet future food demands in a climate change context.

### Barley showed high physiological shade acclimation

The Chl a/b decrease at the lowest irradiance level in barley (50%) showed a typical vegetative response of acclimation to shade, as reported for other species^34^. In this way, these plants are able to extend the wavelength useful range to a shorter wavelength, i.e., the blue fraction. However, wheat did not show any acclimation, in contrast with the results obtained by Dong *et al*.^35^ when studying different light intensities, which were always smaller than the PAR intensities of our study. In addition, the stronger decrease in the Carot/Chl ratio in the 50% irradiance level for barley agrees with the high capacity of acclimation to low light levels previously described for this species^36^. This suggests that barley plants in the full light treatment needed greater photoprotection than plants in the low light environment, in accordance with the photosynthethic parameters of shaded plants. This photoprotection process is partly performed by the carotenoids within the xantophyll cycle, which is known to be related to NPQ, to dissipate excess excitation energy as heat and thus avoid photodamage to PSII^37^. This photoprotection process is also shown in the NPQ parameter in our study, which was found to be higher in barley grown under low irradiance when exposed to high light conditions, revealing that excessive light energy was dispersed through non-photochemical reactions. However, no significant differences were found in wheat in the Carot/Chl ratio nor in the NPQ, even though some authors reported this acclimation for wheat cultivars^38^ in a region with lower solar irradiance intensity than the Mediterranean. Therefore, barley clorophyll pigments helped this species to acclimate to shade, in contrast with wheat, that did not show this adaptative response in our study.

The net photosynthetic light responses curves values for barley and wheat species in full light conditions (R_D_, I_comp_ and I_max_) indicated that barley leaves had a typical shade-leaf response^39^, whereas wheat leaves displayed charasteristic sun-leaf behavior. Furthermore, the higher Φ_(*I*comp-*I*200)_ in barley compared with wheat showed greater photosynthethic efficiency to light capture and carbon fixation at low irradiance levels^40^. All these parameters derived from the light responses curves indicate that barley has potentially better photosynthetic acclimation than wheat to shade conditions.

The fluorescence parameters studied showed that photochemical reactions at high PAR intensities were not as efficient in shaded plants as in full light plants, due to the higher ratio of closed PSII reaction centers caused by excessive incident light in the former. As a result, barley shade-acclimated plants, especially those at 50% irradiance, were less efficient than light-acclimated plants at transferring electrons through photochemical processes in high light availability. To alleviate light stress and avoid photodamage in the PSII antennae of these barley plants, chlorophyll excitation energy was dissipated as heat when exposed to excessive PAR intensities, as revealed by the significant NPQ increase. Therefore, the results obtained from the fluorescence parameters Φ_PSII_, ETR and NPQ in barley indicate that this species developed an efficient shade acclimation strategy through changes in the photosynthetic apparatus, proving that this species is able to adapt its photosynthetic behavior to different light environments^20^. For wheat, no acclimation was found in any of these parameters, in contrast with Zheng *et al*.^38^, who showed shade acclimation in terms of Φ_PSII_ and ETR in some wheat cultivars in a humid-subtropical climate. This could be due to the photosynthetic characteristics of wheat cultivars in each region. The light saturation point reported by Zheng *et al*.^38^ for wheat cultivars was approximately 1000 µmol (photon) m^−2^ s^−1^, while in our study, they saturated by 1800 µmol (photon) m^−2^ s^−1^. The lower saturation point in wheat cultivars could indicate a greater shade-tolerance that could drive low irradiance acclimation in photosynthetic parameters, in contrast the cultivars used in our region.

### Wheat was morphologically better adapted than barley to low irradiance conditions through a major leaf area expansion

Leaf mass area (LMA) is an important plant trait that describes plant strategies to acclimate to varying environmental conditions^41–44^. In most species, LMA is negatively related to light availability. When light is not limitating, plants increase their biomass per leaf area in order to enhance photosynthesis, and conversely, more leaf area per biomass improves light interception unded shaded environments^41^. Our results show this adaptive response in barley and especially in wheat. Plants maximize light capture increasing single leaf surface, but in our study, no significant differences were found in leaf surface in barley between the irradiane treatments, in contrast with the results reported by Zivcak *et al*.^20^. On the other hand, wheat strongly increased its single leaf area in shade, in accordance with Li *et al*.^45^. This different response in the single leaf surface between species regarding low irradiance in our study, reveal that LMA decrease in barley in the reduced light conditions is mostly explained by reduced specific leaf biomass, while in wheat it is justified by larger leaf area. Although it is known that there is no phylogenetically constraint in barley to expand single leaf area^46^, wheat seems to have a greater morphological plasticity to expand leaf surface for increased light capture in reduced light environments in our study.

### Cultivars showed highly different responses to low irradiance, with considerable variability in both barley and wheat

The cultivars responses to grain yield and to different morphophysiological parameters (LMA, Chl (a + b), and NPQ) showed that some barley and wheat cultivars had greater plasticity than others to adapt to lower irradiance conditions through a range of morphophysiological traits. However, our results showed that the amount of variability among cultivars was dependent on the trait considered. When considering the differences in the variability explained by mixed models with and without the cultivar (i.e. between the marginal and conditional coefficient of determination), we observed that the greatest differences were found in the parameters of the light response curve. On the other hand, for morphological parameters, such as LMA, or grain yield, the variability explained by cultivars was less important. These results suggests that selecting the appropriate trait can be an important step during the selection program. Overall, our result indicate that there could be sufficient variability in currently commercialized barley and wheat cultivars to start selection programs based on functional tratis that would help farmers to adopt agroforestry systems.

## Conclusions

To the best of our knowledge, this is the first time that cultivars of winter wheat and barley have been studied and compared in physiological, morphological and production terms to determine whether they are shade-acclimated species with potential for use in agroforestry systems. Our results showed that both wheat and barley increased their grain yield under low irradiance conditions, using different acclimation strategies. Barley showed greater photosynthetic acclimation to shade than wheat, helping to improve physiological performance and therefore grain yield at reduced irradiance levels. Wheat underwent a major morphological acclimation to reach a similar increase in grain yield. Cultivar responses to shade were considerably different in the parameters studied, with some showing high plasticity to shade acclimation and others showing no response. These results reveal that both barley and wheat species are suitable for growing under tree shade in high-radiance climates, highlighting the utility of cultivar selection programs to establish succesful agroforestry practices that could mitigate grain yield decreases caused by foreseeable temperature increases.

## Data Availability

The datasets generated and analyzed during this study are not publicly available given that the CTM2016-80176-C2-2-R project is still yielding results, but are available from the corresponding author on reasonable request.
